# Effects of targeted memory reactivation during sleep at home depend on sleep disturbances and habituation

**DOI:** 10.1038/s41539-019-0044-2

**Published:** 2019-05-02

**Authors:** Maurice Göldi, Björn Rasch

**Affiliations:** Department of Psychology, University of Fribourg, Rue P.-A.-Faucigny 2, CH-1701 Fribourg, Switzerland

**Keywords:** Human behaviour, Human behaviour

## Abstract

Targeted memory reactivation (TMR) during sleep improves memory consolidation. However, it is still unknown whether TMR also benefits memory in real-life conditions. We tested whether TMR during sleep enhances Dutch-German vocabulary learning when applied during multiple nights at home in an unsupervised fashion. During 3 consecutive nights, 66 healthy young participants used an mp3-player to play Dutch words during sleep, without any control of sleep or awakenings by tones (unsupervised TMR). Unsupervised TMR benefitted overall memory scores only in a subgroup of participants, who reported no disturbances by TMR during sleep. Participants who reported general disturbances of sleep showed no benefit, while TMR specifically impaired memory in a third group who reported specific disturbances by the played words during sleep. Separate analysis per night indicated that memory benefits by TMR were significant in the entire sample in the third night only. Our results indicate that sleep disturbances and habituation might be critical factors for the success of unsupervised TMR in a home setting. Habituation to the TMR process as well as automatic sleep monitoring and avoidance of auditory-induced awakenings might be a precondition to successful application of TMR to language learning in real-life.

## Introduction

Re-exposure to memory cues during non-rapid eye movement (NREM) sleep improves later memory performance (e.g.,^1,2^). This technique has been termed “targeted memory reactivation” (TMR).^3,4^ In this technique, sensory or verbal cues are paired with the learning material during an encoding phase before sleep. During subsequent sleep, these cues are presented again during sleep, typically during sleep stages N2 and/or N3. When retrieval is tested after sleep, memory for items that were played during sleep (cued items) is typically better as compared to memory for items not played during sleep (uncued items). Originally, this technique was used to provide evidence for the theoretical assumption that spontaneous reactivation during sleep play a functional role for process of memory consolidation during sleep.^1,5^ According to the active system consolidation hypothesis,^6,7^ spontaneous reactivation of neuronal memory traces in the hippocampus during NREM sleep are essential for strengthening recently acquired memory traces and their integration into cortical long-term memory stores during sleep. The memory benefit of TMR can be explained by selectively biasing these neural reactivation during sleep through cues, resulting in a better consolidated trace.^8,9^ The positive effect of TMR during sleep on memory is now well established and has been shown for a variety of memory cues such as odors,^1,10–14^ sounds,^2,15–17^ melodies^18–20^, or verbal material.^9,21–24^ Based on this numerous and robust empirical evidence, it is now timely to test this technique under real-life settings.

However, all studies cited above have conducted TMR across a single night or nap under well-controlled laboratory conditions including online sleep monitoring by polysomnography. Only one study use olfactory cues to enhance creativity in real life.^25^ Still, it remains to be shown that TMR benefits on memory also occur across multiple nights under unsupervised real-life conditions when factors such as sleep stage, pre-sleep performance level, sound volume or reactivation pausing due to arousal cannot be tightly controlled.

Thus, the aim of this study is to test whether a simple, unsupervised TMR setup applied during sleep improves memory under real-life conditions over consecutive days. As a memory task, we used the Dutch-German vocabulary task for which we have observed memory improvements by TMR applied during sleep in the lab in five independent studies (group size *n* = 15,^21^ *n* = 14, *n* = 13 and *n* = 16^22^ and *n* = 16).^26^

**Fig. 1:**
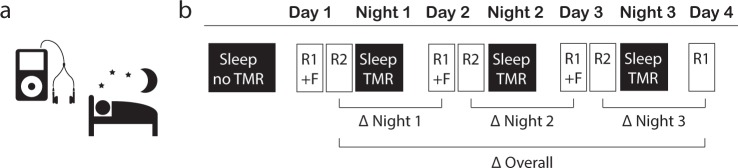
Overview of the procedure. **a** Participants slept at home listening to 60 of 120 Dutch words played over a mp3-player using in-ear headphones. The 60 words played during sleep (cued) wer identical in all three nights. No objective sleep paramteres were recorded (unsupervised targeted memory reactivation (TMR)). **b** Participants performed a Dutch-German word learning task in the evening on four consecutive days. On the first day (Day 1), participants first listened to all 120 Dutch-German word pairs. The Dutch word was presented aurally, and the German translation appeared on the screen (Round 1 + Feedback (R1 + F)). Then, all Dutch words were presented again, and participants typed in the German translation. No feedback was given (R2). On the evening of Day 2 and Day 3, all Dutch words were presented again, and participants typed in the German translation, followed by the correct feedback. (R1 + F). After a short break, all Dutch words were presented again and participants typed in the correct answer, without feedback (R2 + F). On Day 4, participants heard the Dutch words and typed their answer, without feedback (Day 4 R1). Dependent variables are the overall performance changes over the three nights (Δ overall, with performance on Day 1 set to 100%) and performances changes across single nights (Δ night 1, Δ night 2 and Δ night 3, respectively)

A total of 66 healthy young participants were instructed to learn the same 120 Dutch-German word pairs over 4 consecutive days (See Fig. 1, for an overview of the procedure). During the three intervening nights in which participants slept at home in their normal sleeping environment, half of the words (60 Dutch words only) were played (cued) during sleep. The selection of cued words was identical during all three nights of sleep; the other half of the words was never played during sleep (uncued words). For cueing during sleep, participants received an mp3-player containing a sound file starting with 30 min silence and then 1 h of repeatedly playing Dutch words. They were instructed to play the audio file the three consecutive nights when going to sleep. We hypothesized that words played during sleep (cued words) would be better remembered during retrieval after the third night as compared to uncued words. Our results show that TMR during 3 nights of sleep at home generally improved memory for played (cued) words only in a subgroup of participants that did not report sleep disturbances. Participants who reported unspecific sleep disturbances had no memory benefit for cued words by TMR. Furthermore, participants who reported specific awakenings by the words played during sleep even exhibited selectively impaired memory for cued vs. uncued words. Detailed analysis per night revealed that in the entire sample, TMR benefits on memory were significant only across the third night,

## Results

### Overall memory performance

Participants successfully recalled 45.26 ± 1.57 German translations of the 120 newly learned Dutch words before night 1. Four days later at the end of the study procedure they recalled on average 72.58 ± 2.40 words, resulting in a clear improvement of 165.16 ± 4.86% over the multiple learning sessions (Δ overall, with retrieval before the first night set to 100%, *t*_(65)_ = 13.42, *P* < 0.001). The improvement was almost exclusively due to active rehearsal on day 2 and 3 (both *P* < 0.001; see supplementary material and Supplementary Fig. 1). In contrast, memory performance over nights 1, 2, and 3 remained stable relative to their preceding levels (all *P* > 0.05; see Table 1 for an overview of the number of words in each word category and at each measurement point).

**Table 1:** Memory performance in the entire sample (*n* = 66)

	Cued	Uncued	*t*(65)	*P*
*Remembered words (abs.)*				
Day 1 Round 2	22.76 ± 0.79	22.50 ± 0.79	1.58	0.12
Day 2 Round 1 + Feedback	22.73 ± 0.78	23.15 ± 0.85	−0.87	0.39
Round 2	31.47 ± 1.00	31.70 ± 0.98	−0.44	0.66
Day 3 Round 1 + Feedback	31.06 ± 0.96	30.92 ± 1.03	0.24	0.81
Round 2	36.70 ± 1.21	37.91 ± 1.21	−1.95	0.06
Day 4 Round 1	36.15 ± 1.19	36.42 ± 1.28	−0.47	0.64
*Remembered words (rel.)*				
Δ% Overall	163.41 ± 4.66%	167.28 ± 5.61%	−1.14	0.26
Δ% Night 1	101.51 ± 2.23%	105.22 ± 3.10%	−1.50	0.14
Δ% Night 2	100.00 ± 1.72%	98.12 ± 1.72%	1.03	0.31
Δ% Night 3	99.95 ± 1.96%	96.29 ± 1.53%	2.32	0.024^*^
*Gained words (abs.)*				
Δ Overall	15.83 ± 0.69	15.93 ± 0.84	−0.20	0.85
Δ Night 1	4.55 ± 0.34	4.98 ± 0.40	−1.19	0.24
Δ Night 2	4.30 ± 0.28	3.89 ± 0.30	1.28	0.20
Δ Night 3	4.18 ± 0.37	3.42 ± 0.32	2.28	0.026^*^
*Lost words (abs.)*				
Δ Overall	2.44 ± 0.28	2.02 ± 0.30	1.89	0.06
Δ Night 1	4.58 ± 0.35	4.33 ± 0.36	0.83	0.41
Δ Night 2	4.71 ± 0.31	4.67 ± 0.38	0.12	0.90
Δ Night 3	4.73 ± 0.42	4.91 ± 0.43	−0.56	0.57

The table shows number of words for cued and uncued words of each category in absolute (abs.) and relative values (rel.). The total learning list was 120 word pairs, and the words-pairs remained identical over the different trials. For remembered words, relative values refer to the retrieval performance on one retrieval test in each participants, with the individual previous retrieval performance set to 100%. Gains and lost words are calculated with reference to the previous retrieval performance. Mean ± s.e.m are indicated. *P < 0.05

Next, we analyzed memory improvements separately for words cued and not cued during sleep in the three nights of the experiment. In contrast to previous results obtained in the sleep laboratory, we did not observe any general memory benefit of TMR during sleep at home. For cued words, participants increased their memory performance from day 1 to day 4 by 163.42 ± 4.66%, while uncued words improved by 167.29 ± 5.61%. We observed no significant difference in memory improvement between cued and uncued words (*P* > 0.25). Number of cued and uncued words did not differ at baseline (*t*_(65)_ = 1.58, *P* > 0.12, Table 1). The pre-sleep performance at baseline did not correlate with the difference in improvement between cued minus unced words (i.e., memory benefit induced by cueing, *r* = 0.11, *P* > 0.30). Thus, our overall analysis reveals that cueing of Dutch vocabulary during three nights of sleep at home does not generally benefit memory.

**Fig. 2:**
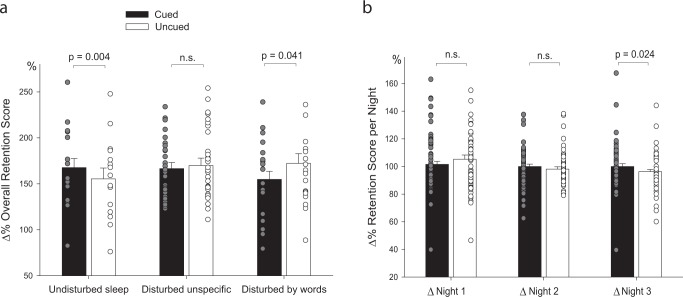
Results. **a** Change in overall memory performance across all three nights with TMR. In undisturbed sleepers (*n* = 15), TMR during sleep at home significantly increased memory for the translation of Dutch words presented during sleep (cued) as compared to words not presented during sleep (Uncued). In participants who reported sleep disturbances unrelated to the words (e.g., discomfort with earplugs etc., *n* = 32), no benefit of TMR was observed. In contrast, in participants who reported awakenings by the words (*n* = 19), TMR impaired memory for cued as compared to uncued words. One outlier in this group is not shown (uncued words: 367%). The impairment remained significant after exclusion of the outlier (*P* = 0.049). **b** Change in performance per night in all participants (Δ night 1, Δ night 2, and Δ night 3, respectively, with performance before each night set to 100%). TMR improved memory for words played during sleep (cued words, black) as compared to words not played during sleep (uncued words, white) only across night 3. n.s. not significant. Means ± standard error of the mean (s.e.m) are indicated

One major difference to previous studies in the sleep laboratory was the unsupervised word presentation during sleep. Thus, the auditory stimulation was not immediately stopped by the experimenter when any signs of awakenings occurred. During the first night, only *n* = 15 participants reported undisturbed sleep during unsupervised TMR. *N* = 32 participants reported unspecific sleep disturbances (i.e., discomfort with headphones, reasons unrelated to TMR). The remaining 19 participants reported that they awakened specifically because of word presentation during sleep. To control for the effects of sleep disturbances, we used the reports from the first night to separate participants in three groups: undisturbed sleepers (*n* = 15), disturbed sleep for unspecific reasons (*n* = 32), disturbed sleep due to word stimulation (*n* = 19). In a 2 × 3 ANOVA, we observed a highly significant interaction between the within-subject factor “cueing” and sleep disturbance group (*F*_(2,63)_ = 5.58, *P* = 0.006, *η*^2^ = 0.15): Over the three nights of stimulation, undisturbed sleepers showed a significant memory improvement by TMR by ca. 12% points similar to previous findings in the sleep lab (cued words: 167.63 ± 1.15% vs. uncued: 155.41 ± 4.41%; *t*_(14)_ = 3.25, *P* = 0.006, see Fig. 2a). Participants who reported unspecific disturbances showed no overall difference between cued and uncued words (*P* > 0.40). In contrast, participants who had reported awakenings by the word stimulation during sleep overall exhibited a significant impairment by TMR by ca. 17% (cued: 154.84 ± 9.78% vs. uncued: 172.30 ± 13.49%, *t*_(18)_ = 2.20, *P* = 0.041). Neither the main effect of cueing (*P* > 0.30) nor group (*P* > 0.80) was significant. Thus, awaking by TMR appears to have an impairing effect on memory consolidation during sleep. Importantly, this impairment seems to be selective for those words presented during sleep, but not for uncued words. Due to the significant interaction of memory performance with auditory-induced awakenings, we present all subsequent results on memory performance also separately for undisturbed sleepers, unspecific disturbances, and disturbances by words (see Table 2, for descriptives of the three groups).

**Table 2:** Descriptives of the three groups

	Undisturbed sleepers	Disturbed (unspecific)	Disturbed by words	*F*(2,63)	*P*
*N*	15	32	19		
Male/female	5/10	10/22	6/13		0.99
Age	21.91 ± 0.81	21.59 ± 0.41	22.26 ± 0.48	0.45	0.64
Sleep quality night 1	2.86 ± 0.22	2.19 ± 0.17	1.79 ± 0.17	6.33	0.003**
Sleep quality night 2	2.67 ± 0.28	2.73 ± 0.12	2.44 ± 0.16	0.90	0.41
Sleep quality night 3	2.89 ± 0.21	2.89 ± 0.10	2.76 ± 0.08	0.25	0.78
Sleep quality without TMR	3.04 ± 0.22	2.91 ± 0.12	3.26 ± 0.10	1.63	0.20
Mean number of restarts night 1	0.80 ± 0.33	0.91 ± 0.20	2.47 ± 0.18	10.17	<0.001***
Mean number of restarts night 2	0.73 ± 0.36	0.50 ± 0.17	1.32 ± 0.30	2.89	0.06
Mean number of restarts night 3	0.27 ± 0.60	0.25 ± 0.09	0.84 ± 0.25	4.39	0.0.016*
Mean number of remembered words before night 1 (baseline)	43.67 ± 3.67	46.28 ± 2.19	44.79 ± 2.93	0.23	0.79

Groups are separated by subjective sleep disturbances reported after night 1. Unspecific sleep disturbances are, e.g., discomfort with the earplugs or other TMR unrelated reasons. The third group reported specific disturbances and/or awakenings by the word stimulation during sleep. Subjective sleep quality was rated on a five-point scale using a subscale of the SF-AR (mean of seven adjectives regarding sleep quality of the previous night)^34^. ^*^P < 0.05; ^**^P < 0.01; ^***^P < 0.001, Mean ± s.e.m are indicated

### Effects of TMR on memory across single nights

In addition to the overall memory effects, we analyzed the effects of TMR on memory for each night separately. For this analysis, memory performance before each night was taken as baseline and set to 100% as in previous studies.^21,22^ In an overall ANOVA using the within-subject factors cueing (cued vs. uncued) and night (nights 1–3), we observed a significant interaction between these factors (*F*_(2,130)_ = 4.01, *P* = 0.03; *η*^2^ = 0.06), confirming that TMR had differential effects on memory for the three nights. In the whole sample, cued words significantly profited from TMR during sleep only in night 3 and were significantly better remembered (99.95 ± 1.96%) than uncued words (96.29 ± 1.53%; *t*_(65)_ = 2.31, *P* = 0.024, Fig. 2b). No cueing benefits occurred across night 1 and 2 (both *P* > 0.14, see Table 1).

In addition, we included the factor “sleep disturbance group” in this analysis. However, we did find neither a main effect of group (*P* > 0.40) nor any two-way interaction with the factor group (all *P* > 0.19). Also, the three-way interaction between the factors “cueing”, “night”, and “sleep disturbance group” was not significant (*P* > 0.40). For reasons of completeness, we report all data inclusive exploratory pair-wise comparisons per night for each sleep disturbance group separately in Supplementary Tables 1–3.

We also calculated the correlation between pre-sleep performance levels and the memory benefit induced by TMR during sleep separately for each night. We observed a negative correlation between pre-sleep performance levels and TMR benefits during night 3 only (*r* = −0.31, *P* = 0.01). No association was observed during night 1 (*r* = 0.12; *P* > 0.30) and night 2 (*r* = −0.20; *P* = 0.11).

Previous studies investigating the effects of TMR on word-pair learning have found word gain/loss analysis to be more sensitive towards the effects of TMR.^21,22,27^ In this study the same overall result emerged when analyzing gained words (words that were not remembered before, but recalled after sleep). In a 2 × 3 ANOVA, we observed a significant interaction for between the factors “cueing” and “night” (*F*_(2,130)_ = 3.50, *P* = 0.033; *η*^2^ = 0.05): Post hoc comparisons revealed that TMR during sleep resulted in an increase in the number of gains for cued words (4.18 ± 0.37) than uncued words in night 3 (3.42 ± 0.32; *t*_(65)_ = 2.28, *P* = 0.026; all other nights *P* > 0.20, Table 1). In the 2 × 3 × 3 ANOVA including also the factor “sleep disturbance group”, we observed no significant interaction including the factor group (all *P* > 0.30). The main effect of group was also not significant (*P* > 0.90). The percentage of lost words (words that were remembered before, but not recalled after sleep) revealed no significant main effects or interaction in the 2 × 3 ANOVA including the factor “cueing” and “night” (all *P* > 0.60, see Table 1, for descriptives and exploratory pair-wise comparisons). Also in the 2 × 3 × 3 ANOVA with the additional factor “sleep disturbance group”, neither main effects nor interaction were significant (all *P* < 0.30, see Supplementary Tables 1–3, for descriptives and exploratory comparisons between gain and uncued gains/losses for each sleep disturbance groups separately).

### Effects of unsupervised TMR at home on sleep quality

Presentation of acoustic cues with the ear phones resulted in a decrease in subjective sleep quality (scaled 0–4) in the first and second night: Participants rated their subjective sleep quality significantly lower in the first experimental night (2.23 ± 0.12) as compared to the night without tone stimulation (3.04 ± 0.08; *t*_(65)_ = 5.70, *P* < 0.001). Sleep quality in night 2 was still significantly reduced (2.65 ± 0.01; *t*_(65)_ = 2.86, *P* = 0.006), while sleep quality in night 3 did not differ from sleeping without sound stimulation (i.e., the night of sleep before start of the experiment (see Fig. 1b), 2.85 ± 0.08; *P* > 0.10). Overall, a linear trend of increasing sleep quality across the 3 experimental nights was observed (*F*_(1,65)_ = 30.29, *P* < 0.001; *η*^2^ = 0.32). In contrast, we observed no significant differences in subjectively reported sleep duration between experimental nights and the control night (experimental nights: 8.07 ± 0.14, 7.71 ± 0.15, and 7.89 ± 0.13 h, respectively, control night: 7.86 ± 0.15 h, all pair-wise comparisons *P* > 0.19).

More specifically, 19 participants reported awakenings and disturbed sleep due to the repeated word stimulation during the first night (15 in the second, 5 in the third night). A total of 32 participants reported awakenings in the first night due to reasons other than the word stimulation (i.e., discomfort with headphones or other unrelated reasons, 29 in the second, and 30 in the third night). Only 15 participants reported undisturbed sleep in night 1 (22 in night 2, 31 in night 3). Subjective sleep quality in night 1 strongly differed between the 3 groups of participants: undisturbed sleepers rated their sleep in night 1 as better (2.86 ± 0.22) as compared to participants reporting unspecific disturbances (2.19 ± 0.17) and participants specifically disturbed by words (1.79 ± 0.17, *F*_(2,63)_ = 8.33, *P* = 0.003, both *P* < 0.02 for post hoc pair-wise comparisons). No difference in subjective sleep quality between the groups occurred during the control night without stimulation (*P* > 0.20). Sleep quality was not correlated with memory benefit induced by TMR (percent remembered cued minus unced words), neither overall nor across a single night (0.14 < *r* < 0.19; all *P* > 0.13).

During the first night, 39 participant restarted the word stimulation at least once, whereas 27 participants reported no restarting (on average 1.33 ± 1.48 times, range 1–7 times). Totally, 20 participants reduced the volume in this night, one increased it. In night 2, 28 participants reported restarting (0.79 ± 1.21 times, range 1–5 times) and 9 a reduction of the volume. In night 3, 19 participants reported restarting (0.42 ± 0.77 times, range 1–3) and 7 a reduction of the volume. The reduction in the number of restarting across the three nights was highly significant (main effect of night: *F*_(2,63)_ = 14.51, *P* < 0.001, *η*^2^ = 0.19). In addition, participants who reported disturbances by the words played during sleep restarted significantly more often (main effect group: *F*_(2,63)_ = 10.84, *P* < 0.001 *η*^2^ = 0.26, both post hoc pair-wise comparisons with sleepers disturbed by words *P* < 0.002). The other two groups did not differ (*P* > 0.80). The interaction between the factor “night” and “sleep disturbance group” was not significant (*F*_(4,63)_ = 2.37, *P* = 0.056, see Table 2, for exploratory comparisons per night). However, the number of restarts was not correlated with the memory benefit by TMR (all *P* > 0.15).

## Discussion

In this study we tested whether unsupervised TMR shows a beneficial memory effect in an uncontrolled environment over multiple days. We have shown that cueing vocabulary of a foreign vocabulary-learning task during the night while sleeping at home shows no memory benefit over a three-night reactivation period. Only after two nights of getting used to the TMR setup, reactivation of Dutch words during sleep improved memory for the German translation in night 3 in all participants, with an effect size similar to previous studies in the sleep laboratory.^20,21^

Due to the unsupervised presentation of word stimuli during sleep, several participants reported awakenings by auditory presentation during sleep. Interestingly, when restricting the analysis to participants that did not report any sleep disturbances, we actually observed a significant overall memory benefit of TMR during sleep for cued over uncued words. In most previous TMR studies in the lab, sleep is continuously monitored and stimulation during sleep is stopped when signs of arousals occur (e.g.,^21^). Alternatively, participants that do not reach a sufficient sleep quality are excluded from these studies (e.g.,^2^). Thus, sleep is undisturbed in most participants of previous TMR studies in the lab. Our study at home suggests that undisturbed sleep might be an important factor for inducing memory benefits by TMR. Still, also in undisturbed participants, this memory benefit was most pronounced in night 3. One possible explanation is that participants needed to generally adapt and habituate to the auditory stimulation procedure including sleeping with the ear phones. Subjective sleep quality only reached normal baseline levels in the third night, which coincides with the TMR benefit on memory. Further studies are needed to more systematically examine the relationship between sleep quality and TMR benefits. An alternative explanation could be that TMR benefits on memory require a certain level of pre-sleep performance levels or prior knowledge. Creery et al.^28^ reported that TMR only benefited memory in participants with high prior learning performance, whereas no TMR effect was observed in low performing participants. TMR during sleep was also ineffective in participants with perfect baseline levels. In addition, Groch et al.^15^ showed that pre-existing knowledge on to-be-learned objects was require for a TMR benefit on memory, while no TMR effect occurred for learning completely unfamiliar object-name associations. As pre-sleep performance levels were quite low before night 1 and consecutively increased across the multiple active retrieval events in our study, one might speculate the learning performance only reached a sufficient level on night 3 to allow for a significant TMR benefit on memory. However, we observed a negative correlation between pre-sleep performance levels and memory benefits induced by cueing in night 3. This result suggests that participants with lower pre-sleep performance might profit more from TMR than participants with higher memory performance, questioning the argument stated above. Further studies are required to test the impact or pre-sleep performance levels on TMR benefits on memory.

In addition or possibly also related to sleep disturbances, habituation to the TMR stimulation and equipment might be highly important for real-life application. Participants who reported sleep disturbances by unspecific reasons (not directly related to the words played during sleep) did not generally profited from TMR during 3 nights of sleep. However, a significant benefit of TMR emerged across night 3 also in this group. Possibly, a longer habituation phase might be required in this group to benefit generally from TMR during sleep.

In contrast, participants who reported auditory-induced awakenings exhibited a selective memory impairment of words played during sleep after the entire experimental procedure. This impairment was most pronounced after the first night during which words were played for the first time. Importantly, memory was only impaired for those words actually played during sleep, while memory for uncued words was unaffected. Thus, it is unlikely that the awakenings generally disturbed consolidation processes during sleep, because in this case also memory for uncued words should have been affected. Interestingly, we have also found this negative cueing effect in a previous study using TMR with mp3 players at home (unpublished observation) and during the piloting phase of the current study. Forgetting processes after reactivation cues have been widely discussed in the context of reconsolidation theory (e.g.,^29,30^). According to this account, reactivating a memory renders the trace again in an active and instable state, which is susceptible to forgetting in the presence of interference. One could speculate that awakening immediately after memory reactivation during sleep represent this type of interference of ongoing reconsolidation processes, thereby leading to a selective forgetting of cued as compared to uncued words (see also ref. ^7^ for a similar argument, however, previous studies find no cueing effect of TMR during wake^19,21,31^). However, we do not have enough data to specifically examine this question, as we do not know when exactly the participants were awakened and what word was played at that time. Future studies are necessary to examine this issue by directly comparing reactivation of words below and above the awakening threshold during sleep.

In general, our findings strongly suggest that sleep disturbances and habituation might be highly important factors that strongly influence TMR effect on memory in a home setting: while undisturbed sleepers generally profit from TMR during sleep, those participants who were specifically disturbed by the words even should specific impairments in memory for the cued words. Note that these effects are differential effects between cued and uncued words. None of the groups was generally better or worse in their memory performance after the 4-day procedure. However, we cannot exclude that also other differences between the current study and previous lab studies might have contributed to our result. While the memory task and the selection of words for TMR was essentially the same as in previous lab studies (see methods, for details), the presentation of the words simply started 30 min after participants had pressed the start button and continued for 60 min. Thus, words could have been played during all sleep stages, including N1 or REM sleep or even waking. In contrast in the sleep laboratory, word presentation is typically started when stable N2 or N3 sleep occurs. In addition, the selection of TMR words remained stable over the three nights. It might be possible that a new selection of TMR words each day would have resulted in higher memory benefits. Finally, we do not know exactly whether the participants actually listened to the words during sleep or not, whether the ear phones stayed it, and how high the exact volume of the words as compared to the background noise in the sleeping environment was. These limitations should be taken into account in future studies.

In sum, we conclude the effects of unsupervised TMR during sleep at home strongly depend on the level of sleep disturbances of TMR and general habituation processes. To effectively enhance memory by TMR outside of the laboratory, future studies should use supervised TMR methodology, i.e., including sleep monitoring and automatic sleep stage detection, automatic stopping of stimulation when sign of awakenings are detected as well as brain-signal based volume control of the sounds. (see e.g.,^32^ for a similar approach developed for closed-loop stimulation of slow-waves).

## Methods

### Subjects

A total of 78 healthy young subjects completed the study, of which 12 were rejected during pre-processing (see Section “Data Pre-Processing”). In all, 66 participants (45 female) between 18 and 30 (21.86 ± 0.30, mean ± standard error of the mean, s.e.m.) years of age were included in all further analysis. All participants had German as their mother tongue and reported no prior knowledge of Dutch or Afrikaans. Participants had no known sleep disorders (screened by the Pittsburgh Sleep Quality Index^33^), dyslexia, learning disorders, or hearing impairments. The internal review board of the University of Fribourg approved the study, and all subjects gave written informed consent prior to participating. Participation was compensated either with 60CHF or university course credits.

### Design and procedure

The experiment included learning and recall sessions in the vocabulary learning task on four consecutive days and auditory cueing during the three intervening nights (See Fig. 1a, b, for a schematic overview of the experimental procedure). On the first day, participants met the examiner in a quiet and undisturbed room between 7 and 9 p.m. They received a set of in-ear sports ear phones (Philips SHQ3200), which they were instructed to use throughout the experiment during the night and the learning/recall tasks. The experiment was done online using LimeSurvey (LimeSurvey GmbH, Hamburg, Germany). On the evening of the second, third and fourth day, participants received an email with a link to the survey at approximately the same time they started the experiment the day before (ca. 7–9 p.m). They were instructed to continue the experiment at home in a quiet and undisturbed setting using the ear phones. In addition, at the beginning of each session participants filled out a questionnaire regarding their previous night’s sleep (SF-A/R,^34^) plus some additional questions about the comfort of the TMR setup. The questionnaire in the first session concerned the night before starting the experiment. As this night was without any sound stimulation, subjective ratings of this night were taken as control night ratings (see Fig. 1b).

### Vocabulary-learning task

The vocabulary-learning task has been previously used to successfully enhance memory during sleep (see ref. ^21^). In this task, participants listen to 120 German-Dutch word pairs. Each Dutch word was presented aurally, and then the written German translation was visible for two seconds in capital letters, followed by a 3-s blank screen. After the first round of learning and a short break, participants continued with the second round during which they listen again to all 120 words and had to type the German translation. No feedback was given. The learning task was identical to our previously used version^21^ with the following exceptions: In our previous study, participants performed an additional learning round with feedback and gave their answers verbally. Also, while an experimenter determined the correctness of the answers in our previous study, here, the correctness of the answer was done automatically (see also Section “Data Pre-Processing”).

On the second, third, and fourth day, participants performed again two rounds on the vocabulary learning task. The first round was identical to the second round on day 1, except that participants received feedback with the correct German translation on screen for two seconds, regardless of the correctness of their answer. The feedback allowed them an opportunity for the acquisition of new word pairs. After a short break on days 2 and 3, the participants did another round of recall without feedback.

For each subject, we therefore have a repetition of the same experiment across three consecutive nights. These repetitions differ from our lab studies, in which performance was tested only once across 3-h of nighttime sleep.^21^

### Reactivation during sleep

Based on recall performance on this first day, 50% of the correctly remembered and 50% of the forgotten Dutch words (60 words) were randomly selected for cueing during the night. We have already successfully applied this stimulus selection procedure in our previous work.^22^ A custom script was used to generate an individual audio file for each participant, which was then loaded onto an iPod (iPod shuffle). The participants then used this same recording for three consecutive nights to present 60 words during their nighttime sleep. Each audio file had a 30-min silent period at the beginning. Then the words selected for cueing during the night were repeated as a block 13 times with a 5-s inter-word silence, totaling roughly 60 min of playback. The first block was presented with linearly increasing volume to minimize waking through sudden sound onset. The last block was presented with linearly decreasing volume. Participants were instructed to start the audio file when they were in bed and ready to go to sleep. Should they wake up from hearing the words, they were instructed to reduce the volume to a comfortable level and restart the audio file.

The stimulation protocol differed from our previous study^21^ as follows: as no experimenter controlled the stimulation (unsupervised TMR), the presentation of words was not restricted to NREM sleep and was not stopped when signs of arousals occurred. No new words were included in the stimulation protocol. In addition, breaks between words were jittered in our previous study (intertrial interval 2.8–3.2 s) and each words was presented approximately 10–11 times.

### Data pre-processing

Prior to further analysis all subjects’ typed answers were checked for spelling mistakes. For the German word “Sonne” (engl. “sun”) the plural form “Sonnen” or small spelling mistakes (e.g., “Soonne”) were accepted as correct. However, when the word resulted in a word similar to another German word (e.g “Sohne”, where “Sohn” would be the correct spelling for engl. “son”), the translation was scored as incorrect. Capitalization was not considered a mistake. This evaluation was done for all given answers by four native German speakers independently, and the results were then aggregated. In some subjects, this procedure unbalanced the number of correct cues chosen for cueing during the night versus uncued words. To avoid any bias, 12 subjects with 4 or more words in one category (cued vs. uncued words) were removed from further analysis, leaving 66 participants.

In the overall analysis, we used performance in the second round of day 1 as encoding performance and performance in the first round of days 4 as final retrieval performance. Relative over retention performance was calculated by dividing the retrieval on day 4 divided by retrieval on day 1, multiplied by 100 (Δ overall). In the separate analysis of each night, the recall round without feedback (second round) of every day was used as the baseline performance for retrieval performance of the next day (first round). We calculate the relative overnight retention performance per night by dividing the retrieval performance on the next day (round 1) with the recall round without feedback on the previous day (round 2). For the three nights, this resulted in three relative overnight retention performances: “Δ Night 1”, “Δ Night 2”, and “Δ Night 3”.

Words that were remembered during the recall were termed as “Remember”. Words that were not remembered during recall were termed as “Non-remember”. For a more fine-grained analysis we further divided our behavioral data into the subgroups “gain”, “loss”, “hithit”, “missmiss”. Gains are words that are not remembered at baseline, but remembered at recall. Losses are words that are remembered at baseline, but not remembered at recall. Hithits are words that are remembered at both baseline and recall. Missmisses are remembered neither at baseline nor recall. Note that the remember group contains the gain and hithit words and the non-remember group contains the loss and missmiss words. We therefore focus only on the gain and loss groups, as the hithit and missmiss groups would give complementary results.

### Statistical analysis

Data were analyzed using *t* tests or repeated-measures analysis of variance (ANOVA) with the within-subject factors “cueing” and the group factor “sleep disturbance group” (“undisturbed sleepers”, “sleepers with unspecific disturbances”, and “sleepers disturbed by words”). For the analysis per night, data were analyzed using the factors “cueing” and “night” or “cueing”, “night” and “sleep disturbance group”. Separate ANOVAs per night were conducted using the factors “cueing” and “sleep disturbance group”. Post hoc pair-wise comparisons were done using two-sided paired *t* tests. Statistical significance threshold was set to *P* = 0.05. Mean values are reported as mean ± standard errors of the mean (s.e.m).

### Reporting summary

Further information on research design is available in the Nature Research Reporting Summary linked to this article.

## Supplementary information

Supplementary Material

Reporting Summary

## Data Availability

Preprocessed data is available on the Open Science Framework at osf.io/auqn4.

## Supplementary information

**Supplementary information** accompanies the paper on the *npj Science of Learning* website (10.1038/s41539-019-0044-2).

## Code availability

Analysis scripts are available on the Open Science Framework at osf.io/auqn4.
