# Supplementary material for: Effects of targeted memory reactivation during sleep at home depend on sleep disturbances and habituation
Source: NPJ Sci Learn. 2019 May 2;4:5. doi: 10.1038/s41539-019-0044-2 (PMC6497651; doi:10.1038/s41539-019-0044-2)
Supplement: Supplementary file 1 — Supplementary Material [file 41539_2019_44_MOESM1_ESM.pdf]

Supplementary Material for

Effects of targeted memory reactivation during sleep at home depend on sleep disturbances and habituation.

Maurice Göldi[1] & Björn Rasch[1*]

[1] University of Fribourg, Department of Psychology, Fribourg, Switzerland

Address correspondence to Björn Rasch, Division of Cognitive Biopsychology and Methods, Department of Psychology, University of Fribourg, Rue P.-A.-Faucigny 2, CH-1701 Fribourg, Switzerland. Email: bjoern.rasch@unifr.ch

Supplementary notes

In addition to the memory retention overnight, we used a similar approach to analyze changes in memory performance across the two learning rounds during the experimental days 2 and 3. Thus, the first recall after sleep was taken as baseline and set to 100%. During this recall, participants received feedback for each word on the correct German translation regardless whether it was correctly recalled or not. Then they were tested immediately afterwards again without any feedback. Overall, we observed a main effect of time (day 2 vs. day 3), with higher relative improvements during active rehearsal on day 2 as compared to day 3 ($F(1,65) = 30.16$, $P < 0.001$, $\eta2 = 0.32$; see Table 1 and Supplementary Figure 1 for absolute numbers of correctly recalled words). No other main effects or interactions were observed (all $P > 0.18$).

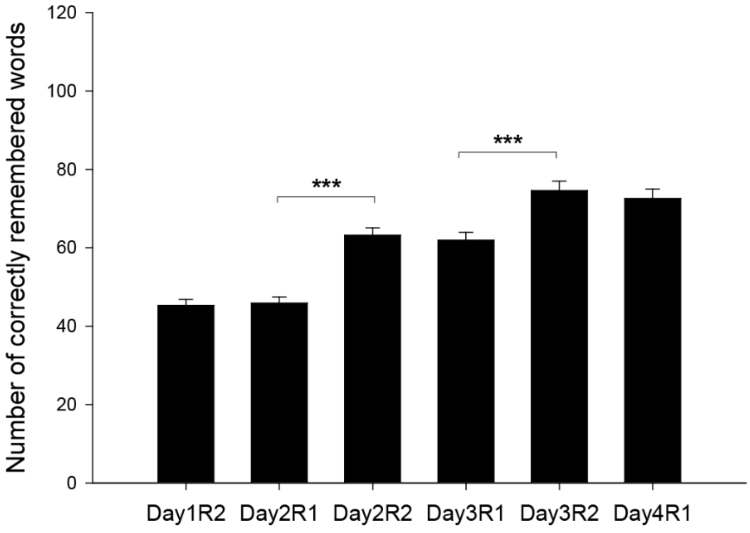

Supplementary Figure 1: Number of words remembered over time. There was an increase during daytime learning (e.g. from the first to the second recall round on Day, Day2R1 to Day2R2), and a stabilization during nighttime retention (e.g., from the second recall round on Day 2 to the first recall round on Day 3, Day2R2 to Day3R1). . ***: P ≤ 0.001, Means ± standard error of the mean (s.e.m.) are indicated.

Supplementary Table 1: Undisturbed sleepers (n = 15)

	**Cued**	**Uncued**	***t*(14)**	***p***
**Remembered words (abs.)**				
**Day 1** **R**ound **2**	21.80 ± 1.75	21.87 ± 1.95	-0.19	.86
**Day 2** **R**ound **1** + Feedback	20.93 ± 1.74	21.20 ± 2.07	-0.26	.80
**R**ound **2**	30.00 ± 1.89	27.80 ± 2.05	1.84	.09
**Day 3** **R**ound **1** + Feedback	29.93 ± 1.55	28.13 ± 2.06	1.73	.11
**R**ound **2**	33.47 ± 2.53	33.07 ± 2.41	.28	.78
**Day 4** **Round 1**	**34.53 ± 1.95**	**32.13 ± 2.47**	**3.08**	**.008****
**Remembered words (rel.)**				
Δ% Overall	**167.63 ± 1.15%**	**155.41 ± 4.44%**	**3.25**	**.006****
Δ% Night 1	98.00 ± 5.06%	101.04 ± 8.98%	-0.51	.62
Δ% Night 2	102.30 ± 4.36%	103.04 ± 4.33%	-0.21	.83
Δ% Night 3	106.38 ± 3.89%	97.13 ± 1.77%	1.96	.07
**Gained words (abs.)**				
Δ Overall	**15.47 ± 1.25**	**12.67 ± 0.84**	**3.42**	**.004****
Δ Night 1	4.27 ± 0.81	4.27 ± 0.75	0.00	.99
Δ Night 2	4.00 ± 0.61	4.27 ± 2.81	-0.55	.59
Δ Night 3	**4.93 ± 0.83**	**3.13 ± 0.62**	**2.22**	**.044***
**Lost words (abs.)**				
Δ Overall	2.73 ± 0.73	2.40 ± 0.97	0.75	.47
Δ Night 1	5.13 ± 1.12	4.93 ± 1.34	0.28	.78
Δ Night 2	4.07 ± 0.59	3.93 ± 0.72	0.17	.87
Δ Night 3	3.87 ± 0.58	4.07 ± 0.66	-0.31	.77

The table shows number of words for cued and uncued words of each category in absolute (abs.) and relative values (rel.). Mean ± s.e.m. are indicated. **: p < 0.01; *: p < 0.05.

Supplementary Table 2: Unspecific sleep disturbances (n = 32)

	Cued	Uncued	*t*(31)	*p*
**Remembered words (abs.)**				
**Day 1** **R**ound **2**	23.25 ± 1.11	23.03 ± 1.08	0.93	.36
**Day 2** **R**ound **1** + Feedback	23.94 ± 0.97	24.13 ± 1.20	-0.25	.81
**R**ound **2**	32.53 ± 1.35	33.28 ± 1.32	-1.18	.25
**Day 3** **R**ound **1** + Feedback	32.25 ± 1.30	32.16 ± 1.36	0.11	.92
**R**ound **2**	38.22 ± 1.61	39.94 ± 1.63	-1.82	.08
**Day 4** **R**ound **1**	37.81 ± 1.48	38.31 ± 1.79	-0.63	.53
**Remembered words (rel.)**				
Δ% Overall	166.54 ± 5.56%	169.89 ± 6.55%	-0.80	.43
Δ% Night 1	105.14 ± 2.95%	106.12 ± 3.68%	-0.31	.76
Δ% Night 2	100.27 ± 2.45%	96.95 ± 1.86%	1.34	.19
Δ% Night 3	**100.09 ± 2.80%**	**96.01 ± 2.37%**	**2.29**	**.029***
**Gained words (abs.)**				
Δ Overall	16.44 ± 0.96	16.97 ± 1.21	-0.75	.46
Δ Night 1	4.88 ± 0.47	5.28 ± 0.60	-0.77	.45
Δ Night 2	**4.56 ± 0.41**	**3.59 ± 0.36**	**2.17**	**.037***
Δ Night 3	4.06 ± 0.57	3.38 ± 0.46	1.55	.13
**Lost words (abs.)**				
Δ Overall	1.88 ± 0.25	1.69 ± 0.28	0.57	.58
Δ Night 1	4.19 ± 0.42	4.19 ± 0.30	0.00	.99
Δ Night 2	4.84 ± 0.42	4.72 ± 0.48	0.23	.82
Δ Night 3	4.47 ± 0.47	5.00 ± 0.57	-1.00	.33

The table shows number of words for cued and uncued words of each category in absolute (abs.) and relative values (rel.). Mean ± s.e.m. are indicated. **: $p < 0.01$; *: $p < 0.05$.

Supplementary Table 3: Sleep disturbed by words (n = 19)

	Cued	Uncued	*t*(18)	*p*
**Remembered words (abs.)**				
**Day 1** **R**ound **2**	22.68 ± 1.49	22.11 ± 1.45	2.00	.06
**Day 2** **R**ound **1** + Feedback	22.11 ± 1.65	23.05 ± 1.43	-1.17	.27
**R**ound **2**	30.84 ± 2.21	32.11 ± 1.85	-1.31	.21
**Day 3** **R**ound **1** + Feedback	29.95 ± 2.20	31.05 ± 2.18	-1.12	.28
**R**ound **2**	36.68 ± 2.52	38.32 ± 2.41	-1.75	.10
**Day 4** **R**ound **1**	34.63 ± 2.74	36.63 ± 2.53	-1.64	.12
**Remembered words (rel.)**				
Δ% Overall	**154.84 ± 9.78%**	**172.30 ± 13.49%**	**-2.20**	**.04***
Δ% Night 1	98.18 ± 4.38%	106.98 ± 5.53%	-1.89	.08
Δ% Night 2	97.74 ± 2.74%	96.22 ± 3.76%	0.38	.71
Δ% Night 3	94.65 ± 3.60%	96.11 ± 3.36%	-0.62	.55
**Gained words (abs.)**				
Δ Overall	15.11 ± 1.52	16.89 ± 1.64	-1.50	.15
Δ Night 1	4.21 ± 0.63	5.05 ± 0.74	-1.25	.23
Δ Night 2	4.11 ± 0.53	4.11 ± 0.66	0.00	.99
Δ Night 3	3.79 ± 0.53	3.74 ± 0.53	0.09	.93
**Lost words (abs.)**				
Δ Overall	**3.16 ± 0.66**	**2.26 ± 0.55**	**2.22**	**.05***
Δ Night 1	4.79 ± 0.50	4.11 ± 0.47	1.95	.07
Δ Night 2	5.00 ± 0.65	5.16 ± 0.87	-0.22	.83
Δ Night 3	5.84 ± 1.12	5.42 ± 1.01	0.97	.35

The table shows number of words for cued and uncued words of each category in absolute (abs.) and relative values (rel.). Mean ± s.e.m. are indicated. **: $p < 0.01$; *: $p < 0.05$.
